# Estimation of Wind Turbine Heights with Shadows Using Gaofen-2 Satellite Imagery

**DOI:** 10.3390/s26041330

**Published:** 2026-02-19

**Authors:** Jiaguo Li, Xinyue Cui, Xingfeng Chen, Hui Gong, Mei Hu, Limin Zhao, Yanping Wang, Kun Liu, Shumin Liu, Yunli Zhang

**Affiliations:** 1 Aerospace Information Research Institute, Chinese Academy of Sciences, Beijing 100094, China; lijg@aircas.ac.cn (J.L.); chenxf@aircas.ac.cn (X.C.); zhaolm@aircas.ac.cn (L.Z.); 2 School of Computer Science and Engineering, University of Emergency Management, Sanhe 065201, China; 23661612@st.cidp.edu.cn (X.C.); wangyanping@cidp.edu.cn (Y.W.); 3 School of Software Engineering, Jiangxi University of Science and Technology, Nanchang 330013, China; 6720231516@mail.jxust.edu.cn (K.L.); liushumin@jxust.edu.cn (S.L.); 9620230006@jxust.edu.cn (Y.Z.); 4 Beijing Institute of Space Mechanics and Electricity, Beijing 100094, China; gonghui2006@163.com; 5 Jiangxi Province Ecological Environmental Monitoring Centre, Nanchang 330039, China

**Keywords:** high-resolution, GF-2 remote sensing image, wind turbine height, shadow

## Abstract

Using high-resolution remote sensing imagery to obtain the wind turbine height is a fast and effective method for monitoring the status of wind turbines after natural disasters such as earthquakes, landslides, and typhoons. A height estimation method tailored for wind turbines is proposed using high-resolution satellite images. First, deep learning techniques are employed to identify wind turbines and extract their shadow information from GaoFen-2 (GF-2) satellite imagery. Specifically, YOLOv5-CBAM and MSASDNet are used for target recognition and shadow extraction, achieving an identification accuracy of 96% and a shadow extraction accuracy of 82.53%. Next, the line-by-line scanning method is applied to remove blade shadow from the whole wind turbine shadow. By calculating the number of pixels occupied by the shadow length of the wind turbine after removing the blade shadow and multiplying by the image resolution, the wind turbine shadow length is obtained. Finally, a spatial geometry model involving the satellite angles, solar angles, and wind turbine shadow length is constructed to retrieve the wind turbine height. An experiment was conducted using GF-2 satellite remote sensing data from a wind farm in Huailai County of China. The actual heights of wind turbines in the estimation area were measured by the field experiment, and the average absolute error was verified to be 2.2 m, demonstrating the effectiveness of the proposed method. The experimental results show that this method can detect the post-disaster status of wind turbines.

## 1. Introduction

Wind energy is an environmentally friendly, sustainable, and limitless renewable source. Governments and organizations worldwide invest in wind energy projects and provide incentives to promote the industry [1]. Wind power is growing more significantly than other renewable energy sources [2] and plays a much more important role in the energy supply system.

According to the 2023 Global Wind Energy Council report, the cumulative installed capacity of new wind turbines globally is expected to reach 680 GW over the next five years [3]. With the increase in wind turbine size and the application of more advanced manufacturing and construction technologies, wind power conversion efficiency has significantly improved. Despite the remarkable development of turbines, their supporting structures still face various challenges. A report from the Caithness Windfarm Information Forum (CWIF) [4] shows that as of 31 December 2016, there were 1999 wind farm accidents, with 54.9% of wind turbine tower collapses related to natural disasters such as typhoons. In August 2015, Typhoon Soudelor severely impacted Taiwan, resulting in the failure of six wind turbines in Taichung [5]. Another severe typhoon Yagi was generated in September 2024 and made landfall along the coast of Wenchang in Hainan Province. At least six wind turbines were damaged [6]. In Asia, the environmental conditions have distinct characteristics that differ significantly from those of other regions like Europe, with the most prominent being high seismic activity [7].

Prowell and Veers [8] reported that wind turbine towers in earthquake-prone areas experience greater seismic loading, sometimes exceeding the wind loading. Under the excitation of a strong pulse-type near-field earthquake, wind turbine may experience local buckling, potentially leading to the collapse of the entire structure [9]. The Kikuno Wind Farm suffered severe damage during the Kumamoto earthquake in 2016, resulting in the No. 2 wind turbine buckling at 13.9 m [10]. Meanwhile, 45.61% of the wind farms were planned without considering land use features and disaster and environmental conditions in Balikesir province of Turkey [11], which faces a great risk of disasters such as avalanches, landslides, and floods.

Natural disasters and the specific distribution of wind farms make it challenging to conduct manual inspections of wind turbines after a disaster. Firstly, typhoons are often accompanied by rain and fog, while landslides are typically triggered by rain or earthquakes [12], which makes it dangerous to reach the disaster site [13]. Significant earthquakes (main shocks) are usually followed by aftershocks [14]. These conditions pose life-threatening risks to the inspector of wind turbines after the disaster. Secondly, visibility and fog are critical for manually inspecting wind turbine damage after a disaster. The World Meteorological Organization (WMO) [15] defines fog as a phenomenon in which horizontal visibility is reduced to less than 1 km due to the condensation of water vapor particles. Wind farms must be located at least 2000 m away from urban areas and at least 500 m from roads during site selection [16]. Additionally, to avoid power generation losses caused by wake effects due to the dense arrangement of wind turbines [17], wind turbines should be arranged perpendicular to the prevailing wind direction, with a row spacing of 3–6 times the rotor diameter of the wind turbine [18]. This results in wind farms covering large areas, with substantial distances between individual turbines. Consequently, post-disaster manual inspections become severely constrained, especially for identifying partially tilted turbines that have not yet undergone complete collapse, as their structural deformations are difficult to discern across such vast distances.

With the advancement of remote sensing technology and artificial intelligence, using satellite imagery to retrieve wind turbine heights has emerged as a novel approach for monitoring post-disaster tower damage. High-spatial-resolution (HSR) satellite images provide extensive coverage and rapid updates of ground conditions, enabling the remote monitoring of height changes for wind turbines before and after disasters. Currently, remote sensing methods for estimating the heights of ground objects rely primarily on three types of sensors: Light Detection and Ranging (LiDAR), Interferometric Synthetic Aperture Radar (InSAR), and high-resolution optical camera [19]. Given the scarcity of existing studies specifically referencing wind turbine height monitoring, this review primarily focuses on the relevant literature concerning building height estimation. LiDAR is an excellent tool for measuring the height of ground objects and is often used as reference and validation data for image-based building height estimation. Park et al. [20,21] proposed a method for classification using sparse, low-density LiDAR point cloud data. This method classifies building point clouds into roof, wall, ground, and high outliers and estimates the building height by extracting only roof points, achieving a height RMSE of 1.32 m. However, the incomplete coverage of spaceborne LiDAR and the high cost of acquiring airborne LiDAR point cloud data make it challenging to rely on LiDAR data for ground object height retrieval. In addition to this data source, Du et al. [22] utilized InSAR technology to obtain Digital Elevation Models (DEMs) for rapid post-earthquake building loss assessment following the 2023 Turkey earthquake. This approach provides an efficient, automated, and large-scale damage assessment method. However, the analysis of individual building damage is constrained by the spatial resolution of the image. Dubois et al. [23] were the first to propose a method for extracting parameters such as the heights of isolated, mid- to high-rise flat-roofed rectangular buildings by analyzing the overlay areas of buildings in InSAR phase images without relying on external information. After removing significant outliers, the accuracy of the extracted height parameters reached 2 m. Sun et al. [24] estimated the heights of individual buildings on a large scale using a single SAR image. By predicting the bounding boxes of buildings, they extracted building heights with an average absolute height error of 4.3 m. However, due to the side-looking imaging geometry and complex backscattering mechanisms, interpreting individual buildings from SAR images is challenging. High-resolution optical imagery, which provides detailed spatial information, can be used for building height analysis and avoids the signal interference issues in SAR technology [25]. Moreover, compared to LiDAR data, optical imagery has lower data acquisition costs. Researchers increasingly use single optical images to explore methods for estimating building heights by analyzing the relationship between buildings and their shadows from various perspectives. Liasis et al. [26] optimized the process of shadow segmentation on satellite images by using custom filters to enhance shadows and reduce intensity inconsistencies. This improved the accuracy of shadow extraction and allowed for building height estimation based on the shadow length, achieving a total variance in building height of 4.13%. Qi et al. [27] proposed a building height estimation method using Google Earth images. By calculating the ratio of building height to shadow length for known buildings, they applied the proposed CSLR method (corner–shadow–length ratio method) to estimate the heights of all other buildings. This method was particularly effective for buildings with heights not exceeding 20 m. Turker et al. [28] utilized the relationship between building shadows and boundaries to detect earthquake-induced variation in building height from post-earthquake aerial images. Using a marker-controlled watershed segmentation method, they achieved an overall accuracy of 80.63% in identifying damaged buildings.

While the above studies on building height estimation are noteworthy, they cannot be directly applied to wind turbines due to significant geometric distinctions. First, unlike rectangular buildings, a wind turbine’s height is defined as the vertical distance from the ground to the top of the nacelle, but its shadow includes rotating blades that can interfere with accurate length extraction. Second, manual intervention is often required to crop targets from large-swath images, such as those from the GaoFen-2 satellite (GF-2) images, before processing, which hinders a rapid post-disaster response.

To address these challenges, this paper proposes an efficient and automated framework for wind turbine height estimation based on high-resolution satellite imagery. To strictly evaluate the model’s accuracy and justify the error margins of the satellite-based estimation, on-site measurements utilizing a laser rangefinder were conducted to serve as a ground truth. The main contributions of this study are summarized as follows:

(1) Unlike conventional building height estimation methods that deal with static, rigid structures, we introduce a novel Scanline-based Blade Shadow Removal Algorithm tailored for wind turbines. This algorithm is capable of eliminating dynamic blade shadows from the turbine shadow, a technical innovation that effectively resolves the specific problem of blade interference affecting height estimation accuracy.
(2) The new systematically comprehensive solution is novel. Unlike previous studies that focused on isolated steps, this study integrates target detection, shadow extraction, and height estimation to form a complete pipeline. This systematic integration itself constitutes an innovation for monitoring wind turbines.

The rest of this paper is organized as follows. Section 2 introduces the relevant information about the GF-2 image dataset used in this study and the methods for acquiring height validation data. Section 3 discusses the deep learning network model used for identifying wind turbines and extracting their shadows, analyzes the geometric features of the wind turbine shadows, and constructs a shadow-based wind turbine height estimation model. Section 4 presents the experimental results and analysis. Finally, Section 5 provides the conclusions and outlines future work.

## 2. Data

### 2.1. Data Source

The GF-2 satellite is a critical component of China’s National Major Science and Technology Project, the “High-Resolution Earth Observation System”. It was successfully launched on 19 August 2014 [29]. GF-2 is equipped with two high-resolution cameras capable of 1-m panchromatic and 4-m multispectral imaging [30]. By optimizing orbital operations and tilt maneuver parameters, the GF-2 satellite achieves a single-camera field of view of 2.1°, resulting in a swath width of 45 km with 2 cameras [31]. Specific sensor technical parameters are shown in Table 1. The GF-2 satellite supports multispectral imaging, covering four spectral bands: blue, green, red, and near-infrared. These bands provide surface information across different spectral ranges, improving feature recognition and meeting the needs of various remote sensing applications.

### 2.2. Dataset

This study selected 113 GF-2 satellite images acquired between 2017 and 2022. The images include diverse terrain, such as plain, mountainous area and coastal regions. The diversity of scene types enhances the generalization and robustness of the wind turbine identification model during its training. The dataset needs to be allocated according to certain principles, with 80% of the data used for the training set and the remaining 20% used for validation to achieve optimal model training results.

Furthermore, preprocessing of the GF-2 images was conducted before their use. Image preprocessing aims to correct the geometric and radiometric distortions in the raw images. Preprocessing of multispectral and panchromatic data includes radiometric calibration, atmospheric correction, and geometric correction, where the geometrically corrected images were uniformly output at a resolution of 1 m. The processed multispectral and panchromatic data were fused to obtain the GF-2 images with a spatial resolution of 1 m for dataset creation. In the above preprocessing, atmospheric correction was performed using the QUantitative and Automatic Atmospheric Correction (QUAAC) method [32], which can efficiently correct HSR satellite images.

Given the inherent regularities in deep learning model data processing, the images and labels must be uniformly cropped and filtered. This process includes discarding samples with irrelevant background values. A total of 9588 samples were prepared, each with a size of 416 × 416 pixels and consisting of green, red, and blue bands. The sample labels for training for wind turbine target recognition and the shadow masks for shadow extraction training were manually interpreted and delineated. Among these, 7670 samples were used for the training set, and the remaining 1,918 samples were used for validation.

Crucially, to ensure the independence of the quantitative height estimation experiments, the wind turbine images used for height validation were selected from a completely different geographical region. These images were strictly excluded from both the training and validation sets of the detection and shadow extraction networks. This spatial separation ensures that the reported accuracy demonstrates the proposed method’s robustness on unseen data.

### 2.3. Validation Data

**Figure 1:**
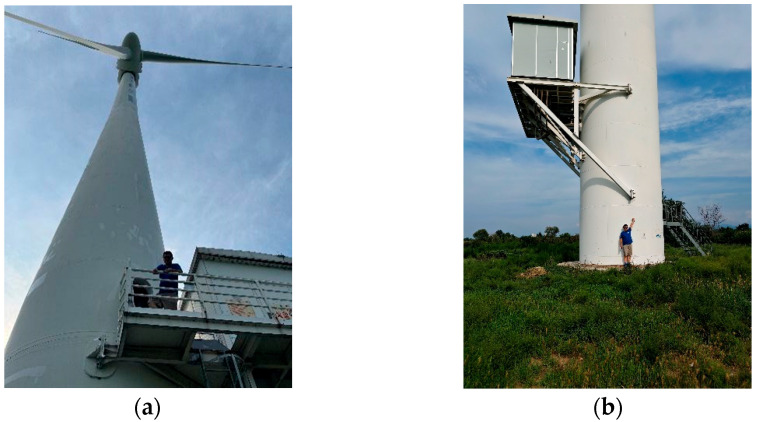
Field measurements of wind turbine height. (**a**) Record the latitude and longitude of the wind turbine near the target turbine; (**b**) keep distance from the root of some of the wind turbines.

The actual heights of wind turbines in the experiments were obtained through field measurements to validate the satellite estimation model proposed in this study. As shown in Figure 1a, the geographic coordinate of each wind turbine was recorded using a Global Position System (GPS) measuring instrument. A laser rangefinder measures the root and top of the wind turbine, and then calculates using the two measurements to obtain the turbine height. It is important to note that when measuring vertical height, a certain distance must be maintained from the object being measured to ensure the accuracy of the laser rangefinder’s measurements (e.g., a distance of about 10 m, as shown in Figure 1b).

**Figure 2:**
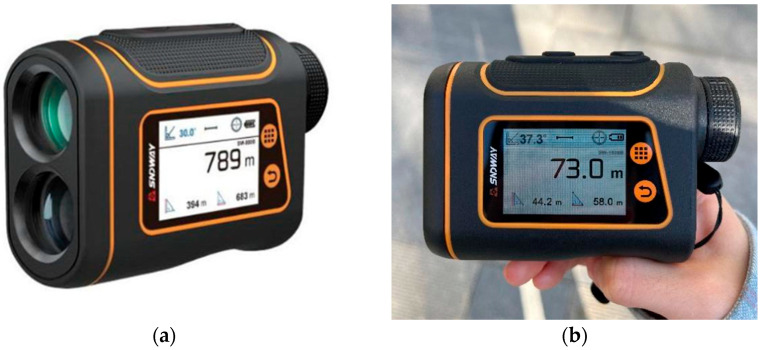
The SW-1500B SNDWAY laser rangefinder telescope. (**a**) The SW-1500B laser rangefinder telescope; (**b**) 73.0 m represents the straight-line distance between the rangefinder and the measured object, 44.2 m represents the vertical distance, and 58.0 m represents the horizontal distance. The data required for height verification is the vertical distance.

The actual height data of the wind turbines were obtained using an SW-1500B laser rangefinder telescope from SNDWAY. As shown in Figure 2, the instrument’s technical specifications are as listed in Table 2. Due to limitations in measurement conditions, the laser rangefinder telescope has a small intrinsic error, and deviations from the field measurement process may be superimposed. Therefore, we adopted a completely consistent ground measurement method to minimize inconsistencies.

### 2.4. Definition of Wind Turbine Height

**Figure 3:**
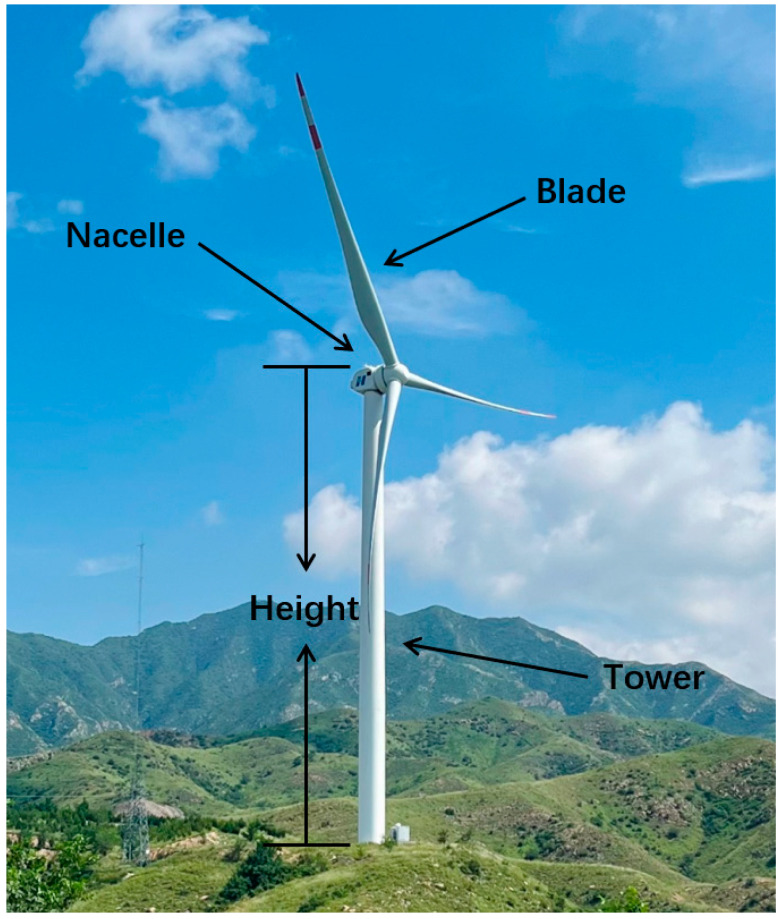
Schematic diagram of the external structure of a wind turbine. The visible external structure consists of the tower, nacelle, and three blades. In this study, the wind turbine height refers to the hub height above the ground, which is the combined height of the tower and nacelle.

The external structure of a typical wind turbine, as shown in Figure 3, consists of blades, a nacelle, and a tower. The height of the wind turbine described in this study refers to the hub height above the ground, which is the tower’s and nacelle’s combined height.

## 3. Method

**Figure 4:**
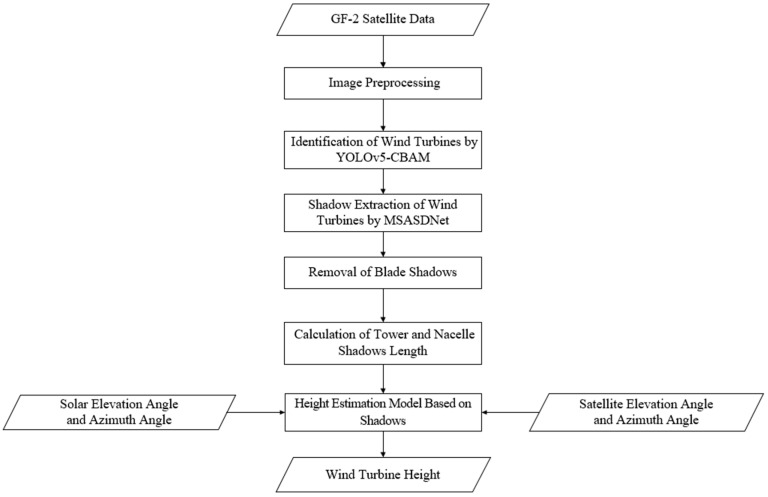
Flowchart of wind turbine height estimation.

This study proposes a height estimation method for wind turbines based on high-resolution image shadows, as illustrated in Figure 4. First, the GF-2 satellite imagery data is preprocessed, and deep learning methods are employed to identify wind turbines in the remote sensing images and extract their shadows. Next, the blade shadows on the wind turbine shadows are removed, retaining only the shadow of tower and nacelle parts. On this basis, the lengths of shadows are calculated. Finally, the wind turbine height is estimated using the lengths of the tower and nacelle shadows, combined with the geometric relationship between shadow and wind turbine.

### 3.1. Wind Turbine Identification

#### 3.1.1. Target Detection Network Basics

Joseph Redmon et al. introduced the YOLO algorithm in CVPR 2016, which presented the first real-time, end-to-end object detection method [33]. It transformed the detection or recognition problem into a regression problem, aiming to find the detection box closest to the actual bounding box of the target using a regression approach. With a single convolutional neural network, YOLO can perform predictions efficiently. Today, the original YOLO algorithm has evolved into versions like YOLOv3, YOLOv4, YOLOv5, YOLOv8, and YOLOv10.

YOLOv5 [34] enhances the model’s modularity and utilizes efficient activation functions to improve the inference speed. By optimizing network layers and components, the model reduces complexity [35] while maintaining a high detection accuracy. It also offers strong customizability [36], making YOLOv5 an ideal choice as the foundational model for deep learning-based object detection in this study.

#### 3.1.2. Convolutional Block Attention Module

The attention mechanism is a way to implement adaptive attention within a network. It allows the network to focus more on effective units during feature extraction while suppressing ineffective features [37]. Convolutional Block Attention Module (CBAM) is a lightweight attention mechanism module that can be easily integrated into existing popular convolutional neural network architectures without additional computational cost. To rescale the original features, it utilizes two pooling methods: max pooling and average pooling [38], which focus on channel information and target location information, respectively. In this study, that mechanism will enhance the network’s focus on wind turbines in the image, highlighting important features of the target, and thereby improving the network’s ability to learn and express features.

#### 3.1.3. YOLOv5-CBAM

**Figure 5:**
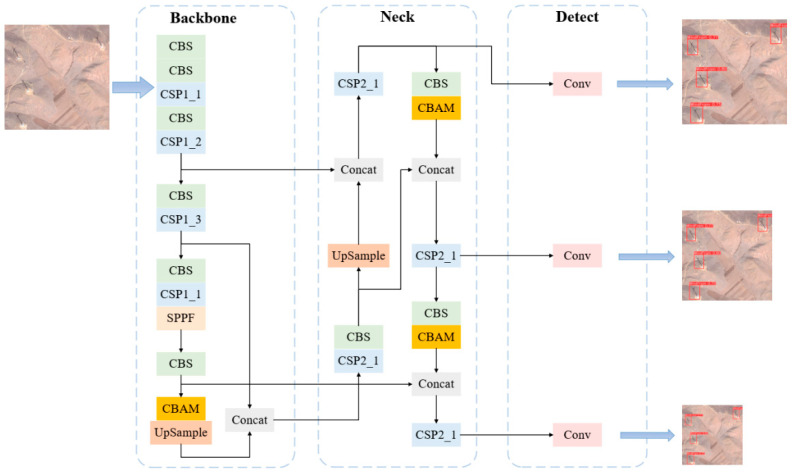
The overall structure of the YOLOv5-CBAM network. On the far-right side of the network structure diagram are the recognition result images of three different sizes output by YOLOv5-CBAM. In the images, the red bounding boxes represent the target area of the wind turbine, and the labels above the boxes indicate the recognition category and confidence level.

Considering the importance of wind turbine target pixels across different channels in red, green and blue bands, the CBAM module is introduced into the YOLOv5 network (YOLOv5-CBAM) as a technical improvement. In the feature pyramid of the neck part of the model, three CBAM modules are added, with the orange modules representing the CBAM module, as shown in Figure 5. The first CBAM module is inserted before the first UpSample module, while the last two CBAM modules are respectively placed before the final two feature Concat modules. These CBAM modules enhance the valid feature information of the wind turbine target and suppress the irrelevant feature information, thereby improving the network’s ability to extract features. The CBAM module can help filter out features more beneficial for target discrimination in the channel dimension. After feature extraction through the backbone network, different channels of the feature map contain multiple dimensional features of the wind turbine target. By enhancing the wind turbine target features of important channels, the model improves the discrimination performance of the wind turbine target. CBAM can generate a spatial attention map based on spatial relationships in the spatial dimension. Considering the spatial relationships between pixels enhances the key spatial features of wind turbine targets and improves the model’s ability to extract semantic information about the targets.

### 3.2. Wind Turbine Shadow Extraction

**Figure 6:**
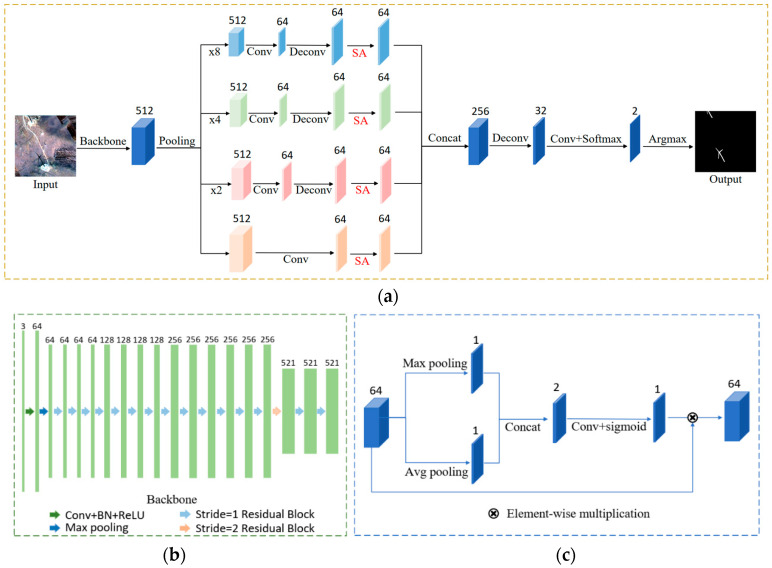
Illustration of MSASDNet. (**a**) The overall architecture of MSASDNet. (**b**) Backbone of MSASDNet. (**c**) Spatial attention nodule.

A shadow detection network based on a multiscale spatial attention mechanism for aerial remote sensing images (MSASDNet) [39] takes the whole shadow image as an input and outputs the shadow mask in an end-to-end manner. The network model consists of three parts, as shown in Figure 6. First, the backbone network is used for initial feature extraction. Then, a multi-scale enhancement feature extraction module based on a spatial attention mechanism (MSA) is applied to extract multi-scale features and suppress the influence of non-shadow areas on the detection results. Finally, a decoder structure combines feature maps of different scales to predict the shadow mask. MSASDNet leverages multi-scale information and spatial attention mechanisms to enhance the network’s ability to extract features from shadow regions while reducing interference from non-shadow areas. As a result, the network can adapt to the detection of shadow regions at different scales, improving its ability to detect small shadow areas and distinguish between low-light and shadow regions.

The backbone of MSASDNet consists of convolution operations, max-pooling, and residual blocks, as shown in Figure 6b. The use of residual blocks helps prevent gradient explosion or vanishing during the training process, which could otherwise leave the network struggling to converge. To better adapt to the detection of shadow regions of different sizes, max-pooling operations of various scales are applied to the feature maps extracted from the backbone network. Convolution kernels of sizes 2 × 2, 4 × 4, and 8 × 8 are used to downscale the feature maps at three different scales, which are then combined with the feature maps at the original scale to form four-scale features. These features are then fused using 1 × 1 convolutions. The convolutional layer is followed by the batch normalization layer and the ReLU activation layer. The feature maps from the three downsampling scales are upsampled through the deconvolution operation. In the deconvolution operation, the kernel size is 4 × 4, the stride is 2, and padding is 1. Finally, to suppress the influence of complex non-shadow areas on the detection results and enhance the detection ability for small regions, the SA module is integrated into the proposed network to obtain multi-scale features. The structure of spatial attention (SA) is shown in Figure 6c.

### 3.3. Wind Turbine Height Estimation

This section describes the wind turbine height estimation method in detail. First, the geometric characteristics of the wind turbine shadow are analyzed. Then, a shadow length calculation method combining the line-by-line blade removal approach and the pixel count method is introduced. Finally, a shadow-based wind turbine height estimation model is established, considering the solar and sensor orientations at different azimuth angles.

#### 3.3.1. Description of Wind Turbine Shadows

The external structure of a typical wind turbine, as shown in Figure 3, consists of the blades, nacelle, and tower, and its shadow is cast by these three components. Since the wind turbine’s blades rotate during operation and the shadow shape varies due to different solar altitudes and azimuth angles at the time of image capture, the wind turbine shadow exhibits different forms.

**Figure 7:**
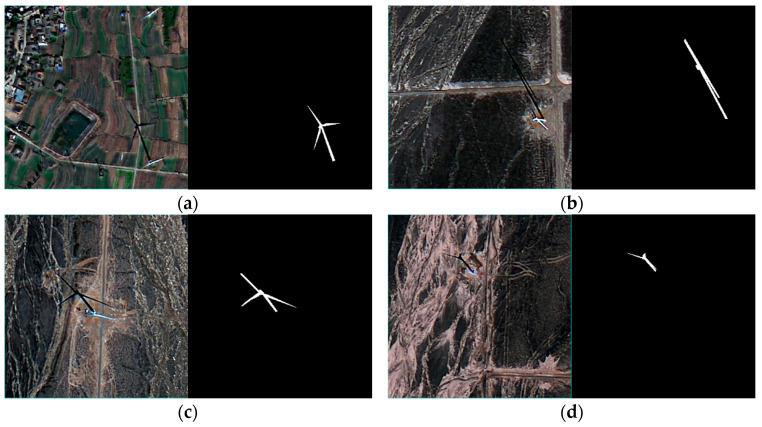
Different geometric shapes of wind turbine shadows. (**a**) The shadow is in an ideal state, with all three blades visible; (**b**) the blade shadows overlap, forming a strip-like shape; (**c**) due to rotation and the effect of the solar altitude angle, the lengths of the blades vary, with one blade’s shadow length exceeding the tower height; (**d**) the shadow only shows the blade at the top of the turbine.

The following is a set of wind turbine images and their corresponding shadow masks. As shown in Figure 7a, the shadow is in an ideal state, with all three blades clearly visible. The ratio of the blade shadow length to the tower shadow length also matches the actual situation. When the solar azimuth changes, the shadow of the same object will change accordingly, as shown in Figure 7b. At this point, the shadows of the three blades overlap, and the shape appears elongated. When the solar elevation angle changes, the length and width of the object shadow also change and are no longer proportional to the object’s actual dimensions, as shown in Figure 7c. The three blades of the wind turbine are of the same length, and the blade length is shorter than the tower height. However, in this case, the shadows of the three blades, due to rotation and the influence of the solar elevation angle, show different lengths, with one blade’s shadow length being longer than the tower’s shadow height. Figure 7d illustrates another scenario of the wind turbine shadow, where only the shadow of one blade is present in the wind turbine’s overall shadow.

The above images depict representative cases; however, the actual shadows of wind turbines vary widely, which adds complexity to the challenge of wind turbine height estimation.

#### 3.3.2. Calculation of Wind Turbine Shadow Length

The shadow length is essential data for height estimation. The combined height of the tower and nacelle describes the height of the wind turbine. Therefore, the premise for estimating the wind turbine height is to calculate the lengths of the tower and nacelle shadows. As described in Section 3.3.1, due to the complex and varying shape of the wind turbine shadow, calculations of the shadow lengths of the tower and nacelle from the overall wind turbine shadow would face interference from the blade shadows. Hence, it is necessary to remove the blade shadows from the wind turbine shadow. Using manual selection for calculation would be time-consuming.

**Figure 8:**
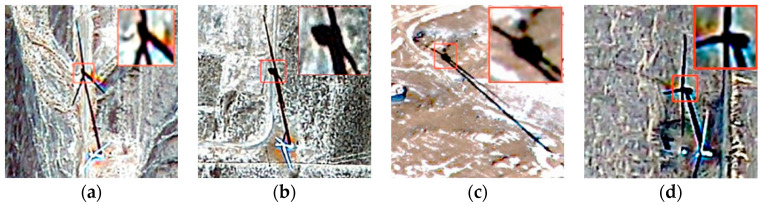
The shadow at the intersection of the blade and the wind turbine nacelle is noticeably wider compared to other parts of the shadow, as shown in the red box in the figure. (**a**) In the ideal shadow state, the shadow at the intersection of the blade and nacelle is wider than the shadow at the base of the blade. (**b**–**d**) In non-ideal states, the shadow at the blade’s and nacelle’s intersection becomes significantly more expansive than the shadow at the base of the blade.

The study of multiple wind turbine shadow images shows that although the overall shape of the wind turbine shadow changes with different solar azimuth and altitude angles, the width ratio between the base and tip of the blades remains unchanged. Additionally, the shadow at the intersection of the three blades and the nacelle becomes significantly more expansive, as shown in the red box in Figure 8. A method is proposed to remove the blade shadow from the wind turbine shadow through line-by-line scanning utilizing the geometric characteristic and the principle of shadow imaging. This method can automatically remove the majority of the blade shadows from the wind turbine shadow, saving a significant amount of time.

Following the extraction of the wind turbine shadow mask, the removal of blade shadows is critical. To address this, we introduce the Scanline-based Blade Shadow Removal Algorithm. To ensure the robustness of the blade shadow removal algorithm across different imaging conditions, we utilize an adaptive threshold multiplier function to determine the segmentation position of the blades. The adaptive threshold multiplier is calculated using Equation (1).

(1) $$\;\lambda =\frac{N_{max}}{N_{initial\;}}$$

where λ denotes the calculated adaptive threshold multiplier. $N_{max}$ represents the maximum number of overlapping pixels recorded during the scan, which represents the width of the blade–nacelle junction or tower height (the scanning process is performed in the horizontal or vertical direction, aligned parallel or perpendicular to the solar azimuth angle). $N_{initial\;}$ denotes the number of overlapping pixels recorded when the scan line first encounters the blade shadow, which represents the physical width of the blade tip.

**Figure 9:**
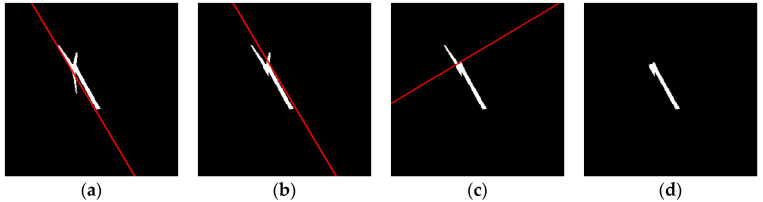
The process of removing wind turbine blade shadows through line-by-line scanning. (**a**) Separation red line for the lower blade; (**b**) separation red line for the upper blade; (**c**) separation red line for the top blade; (**d**) shadow of the tower and nacelle.

The procedure of the Scanline-based Blade Shadow Removal Algorithm consists of the following steps: First, a scan line along the solar azimuth angle is generated. The scanning proceeds pixel by pixel, starting from the tip of the wind turbine blade. When the scan line first intersects the blade shadow, the number of overlapping pixels is recorded as the initial reference width. The scanning continues towards the nacelle until the entire shadow region is traversed. Given that the blade root width is consistently smaller than the tower height, an adaptive thresholding strategy is employed. Specifically, when the number of overlapping pixels first exceeds the product of the threshold multiplier and the initial reference width, the current position is identified as the segmentation point. This line serves as the boundary separating the blade shadow from the tower shadow, as shown in Figure 9a. This step successfully separates the lower blade shadow from the tower shadow. By applying the same methodology in the reverse scanning direction, the upper blade shadow is similarly separated from the tower, as illustrated in Figure 9b. If no scan line position exceeding the threshold pixel count is found during the scan, this indicates that no blade shadow exists on that side of the tower in the image, and no further processing is required. Next, a scan line perpendicular to the solar azimuth angle is generated. The scan line proceeds downward, pixel by pixel, starting from the top of the tower. Leveraging the characteristic that the shadow at the blade–nacelle intersection is wider than both the blade root and the tower, we employ the adaptive threshold multiplier to eliminate blade shadows at the top of the tower, as shown in Figure 9c. If no scan line exceeds the threshold pixel count during this process, it indicates the absence of blade shadows at the tower top, and no further processing is required. Figure 9d displays the isolated tower and nacelle shadows after the complete removal of all blade shadows.

**Figure 10:**
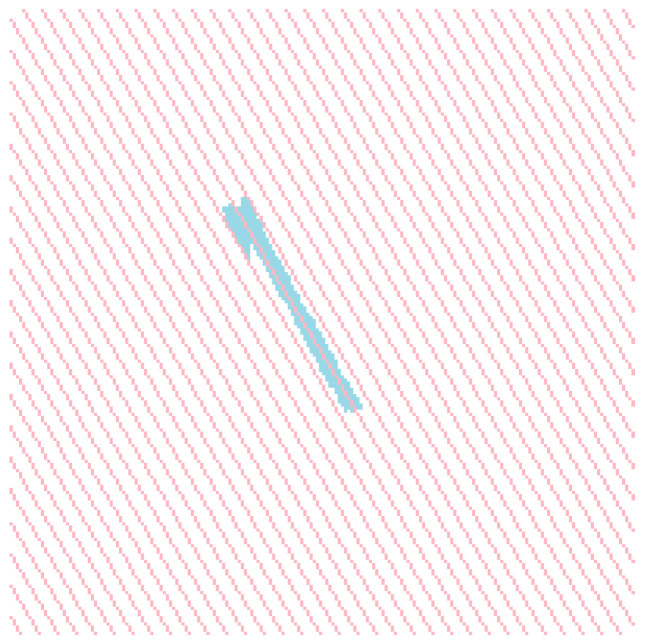
The pixel method calculates the shadow lengths of the tower and nacelle by using the shadow area boundary lines to generate uniform, parallel lines along the solar azimuth direction. These lines are clipped, and the number of pixels overlapping with the shadow area is calculated.

After obtaining the separated shadow of the tower and nacelle, the shadow length can be calculated for height estimation. Existing methods for calculating the shadow length include the pixel method, area and perimeter, corner shortest distance, and fish net. The core of the pixel method is to count the number of pixels covered by the boundary of the shadow area. Specifically, multiple uniform and parallel lines are generated along the direction of the solar azimuth angle, as shown in Figure 10 (for demonstration purposes, the distance between the lines is set to 7 pixels). The shadow region boundary line is used for clipping, and the number of pixels overlapping with the shadow region is counted. The shadow length of the wind turbine is then calculated as the product of the image resolution and the number of pixels. This method suits areas where the measured objects are relatively sparse, shadow regions are regular, boundaries are clear, and the pixel arrangement aligns well with the boundary direction. This aligns with the distribution of wind turbines and the characteristics of turbine shadows after blade removal, making it the chosen method for calculating the shadow lengths of towers and nacelles.

The spatial resolution of the preprocessed GF-2 imagery is 1 m; therefore, the formula for calculating the physical length of the shadow is as follows:

(2) $$L_{physical}=L_{pixel}\;\times \;1$$

During the process of length extraction, irregular edges of the tower and nacelle shadows may result in overlapping lines that are either too long or too short. Using the mean value as the standard for model calculations in such cases can interfere with the experimental results. Therefore, to obtain accurate shadow lengths and building heights, it is necessary to perform gross error detection and removal promptly after generating the overlapping lines. Since the tower and nacelle region after blade removal is relatively regular, the 3σ rule [40] is adopted for gross error removal, with the procedure as follows:

(1) Calculate the standard deviation σ and the arithmetic mean μ of the overlapping line lengths within the shadows of the tower and nacelle.
(2) Calculate the deviation $x_{i}$ of each overlapping line within the shadows of the tower and nacelle from the mean, as shown in Equation (2).
  (3) $$x_{i}=\left|\mu_{i}-\mu \right|$$
(3) Define 3σ as the normal value range with a confidence level of 99.73%.
(4) If $x_{i}\;\leq \;3\sigma$, the overlapping line length is considered a normal value and is retained. If $x_{i}\;>\;3\sigma ,$ the overlapping line length is considered an outlier and is discarded.
(5) Since removing outliers will alter the sample mean and standard deviation, the process needs to be repeated until no more data points are excluded.

#### 3.3.3. Wind Turbine Height Estimation Model Based on Shadows

The angle from true north, measured clockwise, to the direction of the solar ray projection on the ground is called the solar azimuth angle. The satellite azimuth angle is the angle from true north, measured clockwise, to the direction of the satellite’s line-of-sight projection on the ground. The shadow of a wind turbine is a darker area formed in the opposite direction due to its blockage of sunlight. In high-resolution remote sensing images, the visible shadow of the wind turbine does not appear, meaning the wind turbine shadow is either wholly blocked or nonexistent. This includes the following three situations:

(1) When the solar azimuth angle, satellite azimuth angle, and elevation angle are all equal, the satellite cannot observe the wind turbine’s shadow.
(2) When the solar azimuth angle and satellite azimuth angle are the same, and the solar elevation angle is 90°, the wind turbine casts no shadow. In this case, no shadow will be observed in the satellite imagery.
(3) When the solar azimuth angle and satellite azimuth angle are the same, and the solar elevation angle is greater than or equal to the satellite elevation angle, the satellite cannot observe the wind turbine’s shadow.

It is impossible to estimate the wind turbine height based on its shadow in the three situations mentioned above, so these cases will not be discussed.

Based on the relationship between the solar and satellite azimuth angles, the model for estimating the wind turbine height using shadows can be categorized into three types: when the difference between the solar and satellite azimuth angles is greater than 180° when the solar and satellite azimuth angles are the same, and when the difference between the solar and satellite azimuth angles is between 0° and 180°.

**Figure 11:**
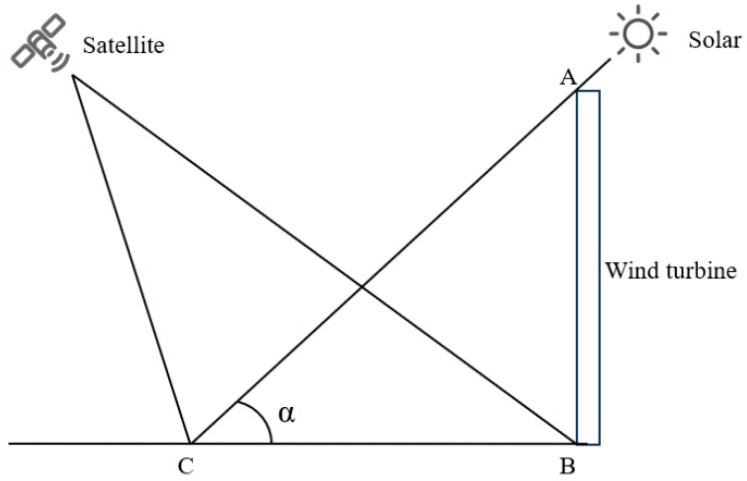
The azimuth angle between the solar source and the satellite is greater than 180°.

When the difference between the solar and satellite azimuth angles is greater than 180°, the satellite is positioned on the opposite side of the building relative to the solar source when acquiring surface information. In this case, the satellite can observe the entire shadow area of the wind turbine, as shown in Figure 11.

Here, AB represents the height of the wind turbine, BC is the shadow length, and α is the solar elevation angle. The wind turbine height can be calculated using Equation (4).

(4) AB=BC × tanα

**Figure 12:**
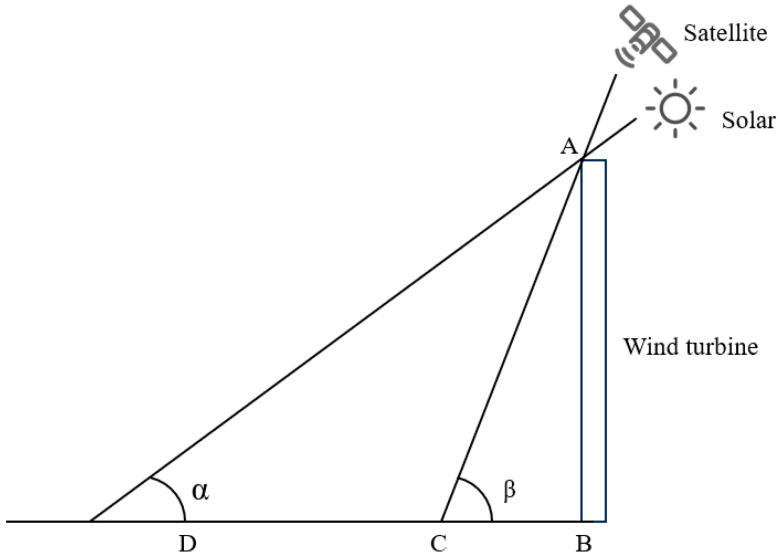
The solar azimuth angle and the satellite azimuth angle are the same.

When the solar and satellite azimuth angles are the same, the satellite and the solar source are on the same side of the wind turbine. In this case, the influence of the azimuth angle does not need to be considered. The relationship between the solar source, satellite, wind turbine, and shadow is shown in Figure 12.

Here, AB represents the height of the wind turbine, BD is the total shadow length, CD is the visible shadow portion, α is the solar elevation angle, and β is the satellite elevation angle. The shadow length measured in the image can be calculated using Equation (5).

(5) $$\mathrm{CD}=\mathrm{BD}-\mathrm{BC}=\frac{\mathrm{AB}}{\tan\alpha }-\frac{\mathrm{AB}}{\tan\beta }$$

According to Equation (5), the formula for calculating the height of a wind turbine can be derived as follows:

(6) $$\mathrm{AB}\;=\;\mathrm{CD}\;\times \;\frac{\tan\alpha \;\times \;\tan\beta }{\tan\beta -\tan\alpha }$$

**Figure 13:**
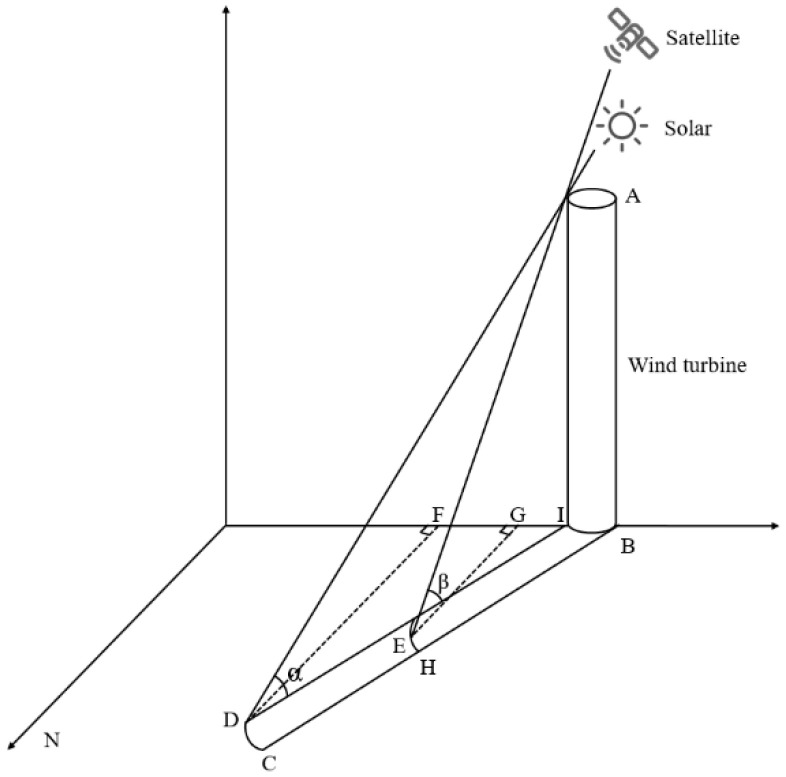
The azimuth angle between the solar source and the satellite ranges from 0° to 180°.

The azimuth angle difference between the solar source and the satellite ranges from 0° to 180°, which is the most common scenario in imagery. The impact of satellite parameters on wind turbine height estimation should be comprehensively considered. At this time, the relationship between the solar source, satellite, wind turbine, and shadow is shown in Figure 13.

α is the solar elevation angle, and β is the satellite elevation angle. DF and HG are auxiliary lines parallel to the true north direction. Rotating clockwise from the true north direction, ∠FDI (obtuse angle) represents the solar azimuth angle, denoted as γ, and ∠GEI (obtuse angle) represents the satellite azimuth angle, denoted as δ. BC represents the actual length of the wind turbine’s shadow, while EC represents the shadow length observed in the remote sensing image. AB represents the height of the wind turbine. The wind turbine height can be calculated using Equation (7).

(7) $$\;\mathrm{AB}=\frac{\mathrm{BC}\;\times \;\tan\alpha \tan\beta \cos\gamma }{\tan\beta \cos\gamma \;-\;\cos\delta \tan\alpha }\;$$

## 4. Result and Analysis

To verify the feasibility of estimating the wind turbine height based on shadow analysis, we selected a specific experimental area to evaluate the effectiveness of the proposed height estimation method. The relative error and absolute error were used as evaluation metrics. GF-2 remote sensing imagery was chosen as the data source for extracting wind turbine heights. The method described in Section 3.3.2 was employed to extract shadow lengths to improve accuracy. Additionally, the actual heights of the wind turbines in the experiment were obtained through field measurements.

### 4.1. Evaluation Indicator

#### 4.1.1. Wind Turbine Identification

The experiment selected important evaluation metrics in the field of object detection to assess the model’s performance, namely Precision (P), Recall (R), Average Precision (AP), Mean Average Precision (mAP), and Accuracy. When evaluating the model size, Precision and mAP are commonly used. The mAP value represents the mean precision metric obtained based on the Precision and Recall of each class in multi-class object detection and serves as a general evaluation metric for object detection tasks.

True Positive (TP) represents the case where a positive sample is correctly predicted as positive. False Negative (FN) represents the case where a positive sample is incorrectly predicted as negative. False Positive (FP) represents the case where a negative sample is incorrectly predicted as positive. True Negative (TN) represents the case where a negative sample is correctly predicted as negative.

P is defined as the ratio of correctly detected targets to the total number of targets detected by the model. The calculation formula is

(8) $$P=\frac{\mathrm{TP}}{\mathrm{TP}+\mathrm{FP}}$$

R is defined as the ratio of correctly detected targets to the total number of actual targets (manually annotated). The calculation formula is

(9) $$R=\frac{\mathrm{TP}}{\mathrm{TP}+\mathrm{FN}}$$

Accuracy is defined as the ratio of correctly predicted samples (including both positive and negative classes) to the total number of samples. The calculation formula is

(10) $$\mathrm{Accuracy}=\frac{\mathrm{TP}+\mathrm{TN}}{\mathrm{TP}+\mathrm{TN}+\mathrm{FP}+\mathrm{FN}}$$

 AP represents the average precision and is used to evaluate the performance of an object detection model for a single class. It is measured by calculating the area under the precision–recall curve at different confidence thresholds. As the confidence threshold changes, precision and recall values vary, forming a curve, and AP is the total area under this curve.

mAP is used to comprehensively evaluate the model’s performance across multiple classes. It is obtained by summing the AP values of all classes and dividing by the total number of classes. mAP provides a more comprehensive assessment of the model’s ability to detect different object categories.

#### 4.1.2. Wind Turbine Shadow Extraction

In semantic segmentation tasks, evaluating model performance is a critical step. The experiment selected important evaluation metrics in the field of semantic segmentation, $\mathrm{Mean}\;\mathrm{Intersection}\;\mathrm{over}\;\mathrm{Union}\;\left(\mathrm{mIoU}\right)$ and $\mathrm{Mean}\;\mathrm{Pixel}\;\mathrm{Accuracy}\;\left(\mathrm{mPA}\right)$, to assess the model’s performance. These metrics effectively reflect the model’s segmentation capability across different categories.

$\mathrm{Intersection}\;\mathrm{over}\;\mathrm{Union}\;\left(\mathrm{IoU}\right)$, also known as the Jaccard Index, measures the similarity between the predicted segmentation results and the ground truth annotations. Its calculation formula is as follows:

(11) $$\mathrm{IoU}\;=\;\frac{\mathrm{TP}}{\mathrm{TP}\;+\;\mathrm{FP}\;+\;\mathrm{FN}}$$

$\mathrm{Pixel}\;a\mathrm{ccuracy}\;\left(\mathrm{PA}\right)$ represents the proportion of correctly classified pixels to the total number of pixels. The calculation formula is as follows:

(12) $$\mathrm{PA}\;=\;\frac{\mathrm{TP}\;+\;\mathrm{TN}}{\mathrm{TP}\;+\;\mathrm{TN}\;+\;\mathrm{FP}\;+\;\mathrm{FN}}$$

mIoU is the average IoU across all classes. In semantic segmentation tasks, mPA is obtained by calculating the PA for all classes and taking the average. Overall, mIoU focuses more on the precision of segmented regions, while mPA emphasizes the overall pixel classification accuracy. Combining both metrics provides a more comprehensive evaluation of the performance of a semantic segmentation model.

#### 4.1.3. Wind Turbine Height Estimation

The experiment selected two commonly used evaluation metrics for assessing the accuracy of height estimation models: $\mathrm{absolute}\;\mathrm{error}\;\left(\mathrm{AE}\right)$ and $\mathrm{relative}\;\mathrm{error}\;\left(\mathrm{RE}\right)$. AE reflects the direct difference between the $\mathrm{predicted}\;\mathrm{height}\;\left(H_{\mathrm{Pre}}\right)$ of the model and the $\mathrm{measured}\;\mathrm{height}\;\left(H_{\mathrm{Mea}}\right)$, representing the deviation of the measurement results from the actual situation. The predicted values of the model are the wind turbine height results obtained from the wind turbines height estimation model based on shadows, while the actual values are the wind turbine height measured on-site for the wind turbines. Their formula is given as

(13) $$\;\mathrm{AE}=\left|{\;H}_{\mathrm{pre}}-H_{\mathrm{Mea}}\;\right|$$

This provides an intuitive measure, indicating the numerical deviation of the model from the proper height, typically expressed in the same units, such as meters or centimeters.

RE describes the proportion of model error relative to the true value. It is the ratio of absolute error to the true value and is often expressed as a percentage:

(14) $$\mathrm{RE}\;=\;\left(\frac{\mathrm{AE}}{H_{\mathrm{Mea}}}\right)\;\times \;100\%$$

RE provides a normalized measure of error, allowing the magnitude of the error to be compared against the actual value. This facilitates the evaluation of model accuracy across different scales or units. RE can more clearly demonstrate the model performance under various conditions, making it particularly suitable for scenarios where multiple models need to be compared across different data ranges. In this study, these two metrics comprehensively assess the accuracy and reliability of the height estimation model, offering valuable insights for further model optimization.

### 4.2. Experimental Results and Analysis

#### 4.2.1. Wind Turbine Identification

The YOLOv5-CBAM network is compared with the two-stage classic model Faster RCNN and the YOLOv7 network. The Faster RCNN network uses ResNet as the backbone, and YOLOv7 uses the pre-trained weights of YOLOv7x.pt. The input image size is 416 × 416, and the other parameters are the same for both models.

To ensure the reproducibility of the experimental results, this paper details the model training environment and key parameter settings. All experiments were conducted on a workstation equipped with an NVIDIA GeForce RTX 4060 GPU (8 GB VRAM) and a 13th Gen Intel^®^ Core™ i7-13700H processor. The deep learning framework used was PyTorch. The model was trained for 500 epochs, with a batch size of 16 and an initial learning rate of 0.01. Furthermore, to enhance the model’s generalization ability, the Mosaic data augmentation strategy was employed during the training phase. The trained weights are used to test the test set, and the recognition accuracy is shown in Table 3.

From the values of the metrics in the table, it can be observed that, compared to the Faster RCNN and YOLOv7 networks, YOLOv5 outperforms these two models in all metrics. For example, YOLOv5 achieves a precision of 0.957, while the other models are around 0.93. This indicates that YOLOv5 is more suitable for recognizing high-resolution remote sensing wind turbine targets. After introducing the CBAM attention mechanism, all the metrics showed improvement. This suggests that the CBAM attention mechanism enhances the wind turbine features in the Neck part of the YOLOv5 network, which benefits the network’s feature extraction and learning of wind turbine characteristics.

#### 4.2.2. Wind Turbine Shadow Extraction

The MSASDNet network is compared with the classic models Deeplabv3+ [41], PSPnet [42], and SwinUnet [43]. The input training images have a size of 416 × 416, and the other parameters are set to be the same for training.

All experiments were conducted on a workstation equipped with an NVIDIA GeForce RTX 4060 GPU (8 GB VRAM) and a 13th Gen Intel^®^ Core™ i7-13700H processor. The deep learning framework used was PyTorch. The model was trained for 350 epochs with a batch size of 8 and an initial learning rate of 0.001, using the Adam optimizer. Furthermore, to enhance the training data, an Overlapping Sliding Window Cropping strategy was employed during the training phase. After training, the obtained weights are used to test the test set, and the prediction accuracies are shown in Table 4.

From the table above, it can be seen that MSASDNet outperforms the other three deep learning methods across all metrics. For example, the mIoU scores of the other three methods are all below 65. PSPNet has an mPA score of 62.58, while the mPA scores of the other two deep learning methods are 75.23 and 77.31, respectively. Clearly, MSASDNet performs the best in shadow detection.

#### 4.2.3. Height Estimation Experiment and Analysis

**Figure 14:**
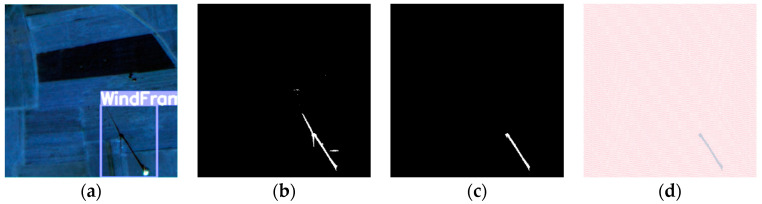
The process of wind turbine height estimation. (**a**) Identification of wind turbines by YOLOv5-CBAM. (**b**) Extraction of wind turbine shadows by MSASDNet. (**c**) Removal of wind turbine blade shadows. (**d**) Removal of wind turbine blade shadows.

The experimental area consists of a GF-2 remote sensing image with a spatial resolution of 1m, located in Hebei Province, China. At this time, the azimuth difference between the solar source and satellite exceeds 180 degrees. Taking a single wind turbine as an example, the visual results of the wind turbine recognition, shadow extraction, and shadow length calculation processes are shown in Figure 14. First, the YOLOv5-CBAM model was used to identify wind turbines within the GF-2 remote sensing image, with the results shown in Figure 14a. Second, the MSASDNet model was applied to extract the shadows of wind turbines, as illustrated in Figure 14b. Finally, the line-by-line scanning method was used to remove the blades, and the shadow length of the wind turbine tower was calculated using the pixel count method, as shown in Figure 14c,d. With the shadow lengths of the tower and nacelle obtained, the shadow-based wind turbine height estimation model constructed in Section 3.3.3 was used. The estimated height of the wind turbine was calculated to be 61 m.

The actual heights of the wind turbines in the experimental area were obtained through field measurements and compared with the calculated heights. The error curve of the height estimation results for the wind turbines in the experimental area was plotted, as shown in Table 5.

The absolute error of the wind turbine is as small as 0.2 m. The estimation results show that incomplete blade removal causes more significant absolute errors. Overall, the average absolute error of the wind turbine height extracted by this method is 2.2 m, which meets the requirements for wind turbine detection before and after natural disasters and demonstrates practical value.

### 4.3. Error Analysis

The accuracy of wind turbine height estimation primarily depends on the quality of shadow detection and the extraction results of the shadow lengths of the turbine tower and nacelle. Through analysis, the factors influencing the accuracy of wind turbine height estimation can be categorized as follows:

(1) Shadows projected onto bright surfaces, such as bare land, roads, or water, can cause significant changes in shadow spectral characteristics, affecting detection results. When shadows are cast on darker surfaces, such as vegetation shadows, the shadow areas may become excessively large, making them difficult to distinguish. Additionally, the shadow edges may become irregular. These situations may hinder the model’s ability to effectively differentiate shadows from other features, adversely affecting extraction results and causing subsequent calculation errors.
(2) The premise of calculating the wind turbine height based on shadows in this study is that the ground where the turbine and its shadow are located is flat, and the shadow information is complete. Suppose the shadow is cast on uneven terrain. In that case, it may disrupt the geometric functional relationship between the turbine and its shadow, interfere with shadow detection results, and subsequently affect the height calculation results.
(3) Errors may occur when removing wind turbine blade shadows, either by failing to remove them entirely or by excessively removing other shadow regions. These issues can affect the subsequent calculation of the tower shadow length, thereby impacting the height estimation results.
(4) A higher spatial resolution enables a more precise extraction of wind turbine shadow areas, resulting in more accurate measurements of tower shadow lengths.
(5) Poor weather conditions, such as haze, can slightly interfere with laser rangefinders. Additionally, inaccuracies in identifying the highest and lowest points of the wind turbine during field measurements can impact the height estimation accuracy.

## 5. Conclusions

Although many studies have focused on estimating heights using shadows, they primarily target regular buildings and have not explored height estimation for wind turbines. In this paper, a fully automated method for estimating wind turbine heights was developed and validated in an experimental area to confirm the effectiveness of the proposed framework. First, deep learning techniques were employed for wind turbine target recognition and shadow extraction, enhancing the efficiency of shadow-based height estimation. Second, a combination of the line-by-line blade removal method and the pixel count calculation method addressed the interference of blade shadows during the automated extraction of tower and nacelle shadow lengths. Finally, by comparing with the actual heights of wind turbines measured by the field experiment, the model’s estimated wind turbine height was validated, with an average relative accuracy of 3%, demonstrating the effectiveness of the proposed method. By automating the estimation of wind turbine heights, the proposed approach assesses turbine conditions and broadens shadow-based height estimation application scenarios.

However, due to the complexity of the automated height estimation process and the current lack of specific consideration of terrain impact, the estimated height results contain a certain degree of error. Furthermore, the verification of the wind turbine height is currently limited by the fact that obtaining precise physical height values requires manual on-site field measurements, a process severely constrained by geographical accessibility and labor intensity. Additionally, the current reliance on a single satellite limits the temporal resolution required to capture short-duration destruction events, which could be addressed by employing satellite constellations. Consequently, when applied in disaster relief scenarios, the proposed method is best utilized for providing preliminary identification and calculation results, which can ultimately be confirmed through manual visual interpretation. Future research will focus on three key areas to address these limitations: first, conducting in-depth research on wind turbines located in complex terrains to mitigate environmental interference; second, further optimizing the blade separation algorithm to improve the segmentation performance; and third, actively expanding the field validation dataset to include a broader range of turbine models and terrain conditions. A larger sample size will allow for a more comprehensive statistical analysis of error sources, enabling further refinement of the model’s robustness and generalization capabilities.

## Figures and Tables

**Table 1:** Technical parameters of GF-2 satellite sensor.

Parameters	Panchromatic/Multispectral Camera	
Spatial Resolution	Panchromatic	1 m
	Multispectral	4 m
Spectral Band	Blue: 0.45–0.52 µm;	
	Green: 0.52–0.59 µm;	
	Red: 0.63–0.69 µm;	
	NIR: 0.77–0.89 µm	
Swath Width	45 km (combined with 2 cameras)	
Revisit Cycle (with side-swing)	5 days	

**Table 2:** Technical parameters of the laser rangefinder for field measurements.

Product Parameters			
Range Error	±(1.0 m + D × 0.3%)	Field of View	7.0° ± 5%
Telescope Magnification	6X ± 5%	Diopter Adjustment Range	±6° diopter
Telescope Objective Aperture	23 mm	Angle Measurement Range	±90°
Telescope Eyepiece Aperture	15.0 mm	Measurement Units	m (meters)/Y (yards)

**Table 3:** Recognition accuracy of test set for different network models.

Method	P	R	AP@0.5	AP@0.5:0.95
Faster RCNN	0.934	0.943	0.937	0.675
YOLOv7	0.935	0.944	0.938	0.670
YOLOv5	0.957	0.946	0.953	0.744
YOLOv5-CBAM	0.960	0.949	0.957	0.746

**Table 4:** Prediction accuracy of the test set for different network models.

Model	PSPnet	DeepLabV3+	SwinUnet	MSASDNet
*mIoU*	57.83	64.95	62.89	74.91
*mPA*	62.58	75.23	77.31	82.53

**Table 5:** Height estimation results of wind turbine.

Wind Turbine Number	Measured Height (m)	Estimated Height (m)	Absolute Error (m)	Relative Error (%)
1	63.1	65	1.9	3.01
2	64.2	61	3.2	4.98
3	64.6	62	2.6	4.02
4	77.3	75	2.3	2.98
5	75.5	77	1.5	1.99
6	76.0	74	2.0	2.63
7	75.2	77	1.8	2.39
8	75.2	75	0.2	0.27
9	72.7	75	2.3	3.16
10	79.2	75	4.2	5.30

## Data Availability

The data presented in this study can be made available following reasonable request to the corresponding author.
